# Revision anterior cruciate ligament reconstruction restores knee laxity but shows inferior functional knee outcome compared with primary reconstruction

**DOI:** 10.1007/s00167-018-5059-3

**Published:** 2018-07-17

**Authors:** Riccardo Cristiani, Björn Engström, Gunnar Edman, Magnus Forssblad, Anders Stålman

**Affiliations:** 1 Department of Molecular Medicine and Surgery, Stockholm Sports Trauma Research Center, Karolinska Institutet, Stockholm, Sweden; 2 Capio Artro Clinic, Sophiahemmet Private Hospital, Valhallavägen 91, 11486 Stockholm, Sweden

**Keywords:** Anterior cruciate ligament, Primary ACL reconstruction, Revision ACL reconstruction, Knee laxity, KOOS

## Abstract

**Purpose:**

To evaluate and compare knee laxity and functional knee outcome between primary and revision anterior cruciate ligament (ACL) reconstruction in the same cohort of patients.

**Methods:**

Patients who underwent primary and revision ACL reconstruction (ACLR) at Capio Artro Clinic, Stockholm, Sweden, from 2000 to 2015, were identified in our local database. Inclusion criteria were: same patients who underwent primary hamstring tendons (HT) and revision bone–patellar tendon–bone (BPTB) autograft ACLR, no associated ligament injuries and no contralateral ACL injuries/reconstructions. The cause of revision ACLR was graft rupture for all patients. The KT-1000 arthrometer, with an anterior tibial load of 134-N, was used to evaluate knee laxity preoperatively and 6-month postoperatively. The Knee Injury and Osteoarthritis Outcome Score (KOOS) was collected preoperatively and at the 1-year follow-up.

**Results:**

A total of 118 patients with primary and revision ACLR arthrometric laxity measurements were available (51.0% males; mean age at primary ACLR 21.7 ± 7.1 years and revision ACLR 24.3 ± 7.5 years). The mean preoperative and postoperative anterior side-to-side (STS) difference values were not significantly different between primary and revision ACLR. However, primary ACLR showed a significantly higher frequency of postoperative anterior STS difference > 5 mm compared with revision ACLR (8.4 vs 5.0%; *P* = 0.02). The KOOS was available for primary and revision ACLR for 73 patients (55.4% males; mean age at primary ACLR 21.6 ± 7 years and revision ACLR 24.7 ± 7.3 years). Preoperatively, revision ACLR showed significantly higher scores in all KOOS subscales, except for the activity of daily living (ADL) subscale. For the primary ACLR, the improvement from preoperatively to the 1-year follow-up was significantly greater in all KOOS subscales and, the postoperative scores were superior for Pain, ADL and Sports subscales compared with revision ACLR.

**Conclusions:**

The findings of this study showed that anterior knee laxity is restored with revision BPTB autograft ACLR after failed primary HT autograft ACLR, in the same cohort of patients. However, revision ACLR showed a significantly inferior functional knee outcome compared with primary ACLR. It is important for clinicians to inform and set realistic expectations for patients undergoing revision ACLR. Patients must be aware of the fact that having revision ACLR their knee function will not improve as much as with primary ACLR and the final postoperative functional outcome is inferior.

**Level of evidence:**

Retrospective cohort study, Level III.

## Introduction

The number of anterior cruciate ligament (ACL) reconstructions significantly increased in recent years [19]. The annual incidence of primary ACL reconstruction (ACLR) is reported to be 34–38/100,000 inhabitants in Norway and Denmark [9, 23]. ACLR is very successful in restoring knee laxity and improving subjective knee function [5, 14]. However, the 7–10% failure rate of primary ACLR [11] highlights the problem of revision surgery. Data from the Danish knee ligament reconstruction registry showed a revision rate for primary ACLR of 3% 2 years after surgery [23] and of 4.1% 5 years after surgery [22]. According to Paterno et al. [30], an athlete in the age between 10 and 25 years who undergoes ACLR has a 15 times greater risk of being injured again in the same knee compared to an athlete with a healthy knee, during the first 12 postoperative months.

Several studies suggested that revision ACLR produces inferior results compared with primary ACLR [6, 16, 20, 28, 29, 37]. However, these studies are based on a matched group analysis, including different patients for primary and revision ACLR. Moreover, they lack of homogeneity, including different graft types for both surgeries.

Patients undergoing revision ACLR need a thorough counseling regarding their expectations after surgery. To study the same cohort of patients would accurately determine the outcome after revision ACLR in comparison with primary ACLR. These findings could help clinicians to inform and set realistic expectations for patients undergoing revision surgery.

The purpose of this study was to evaluate and compare knee laxity and functional knee outcome between primary hamstring tendons (HT) ACLR and revision bone–patellar tendon–bone (BPTB) autograft ACLR, within the same cohort of patients. The hypothesis was that revision ACLR restores knee laxity but is associated with inferior functional knee outcome compared with primary ACLR.

## Materials and methods

Patients who underwent primary and revision ACLR at Capio Artro Clinic, Stockholm, Sweden, from 2000 to 2015, were identified in our local database. Inclusion criteria for this study were: same patients who underwent primary HT autograft ACLR and revision BPTB autograft ACLR, no associated ligament injuries and no contralateral ACL injuries/reconstructions. The cause of revision ACLR was graft rupture, due to a new trauma, for all patients.

A total of 200 patients, with isolated primary HT autograft ACLR and revision BPTB autograft ACLR, met the inclusion criteria. Two study cohorts were generated after applying the exclusion criteria. A first cohort, for the comparison of knee laxity, was established after excluding patients with time from primary ACLR to ACL graft rupture < 6 months before arthrometric evaluation (*n* = 42) and patients with missing KT-1000 values for primary or revision ACLR (*n* = 40). A second cohort, for the comparison of functional knee outcome, was established after excluding patients with time from primary ACLR to ACL graft rupture < 12 months before KOOS collection (*n* = 97) and patients with missing KOOS values for primary or revision ACLR (*n* = 30).

### Surgical technique and rehabilitation

All patients were operated using a single-bundle technique for both primary and revision reconstruction. Primary ACLR was performed using quadrupled semitendinosus or semitendinosus–gracilis tendons autograft. The HT autograft was fixed with an Endobutton fixation device (Smith & Nephew, Andover, Mass, USA) on the femoral side and Ultrabraid (Smith & Nephew, Andover, Mass, USA) or Ethibond no. 2 sutures (Ethicon Inc., USA) tied over an AO bicortical screw with a washer on the tibial side or using an interference screw (RCI, Smith & Nephew, Andover, Mass, USA). Ipsilateral BPTB autograft was used for revision ACLR. The graft was fixed with an Endobutton fixation device (Smith & Nephew, Andover, Mass, USA) or an interference screw (Softsilk, Smith & Nephew, Andover, Mass, USA) on the femoral side and with an interference screw on the tibial side (Softsilk, Smith and Nephew, Andover, Mass, USA). Removal of the old hardware was performed when this compromised the drilling of the new tunnels or the graft fixation. No additional surgical procedures, such as lateral extra-articular tenodesis or antero-lateral ligament reconstruction, were performed in any case of primary or revision ACLR.

The patients followed a standardized rehabilitation protocol after both primary and revision ACLR. Quadriceps strengthening was restricted to closed kinetic chain exercises during the first 3 months. Return to sports was allowed at earliest after 6 months.

### Evaluation

The KT-1000 arthrometer (MEDmetric, Corp., San Diego, CA, USA), with an anterior tibial load of 134-N at 20° of knee flexion, was used to evaluate anterior knee laxity preoperatively and 6 months postoperatively for primary and revision ACLR. At least three measurements for each knee were made and the median value was registered. All tests were performed by experienced physiotherapists at our outpatient clinic. The anterior tibial translation (ATT) reduction from preoperative to postoperative for the ACL-reconstructed knee and the preoperative and postoperative difference in displacement (side-to-side, STS, difference) between the ACL-injured knee and the healthy knee were expressed in millimetres. Postoperative STS difference values were stratified according to the IKDC knee examination form [12] in three groups: ≤ 2, 3–5, and > 5 mm. “Surgical failure” was defined as a STS difference greater than 5 mm (IKDC grade C and D).

The functional knee outcome was evaluated using the Knee Injury and Osteoarthritis Outcome Score (KOOS) [31–33], collected preoperatively and at the 1-year follow-up for both primary and revision ACLR. The KOOS is a frequently used disease-specific patient-reported outcome measure for measuring subjective outcome in patients with ACL injury and ACLR [13]. It is divided in five subscales: Pain, Symptoms, Activity of Daily Living (ADL), Function in Sport and Recreation, and Knee-related Quality of Life (QOL). A score of 0 represents the worst possible outcome while 100 is the maximum score for a subscale.

The number of chondral injuries and associated meniscal procedures (resection and repair of medial and lateral meniscus) for both primary and revision ACLR was also reviewed.

This study was approved by the Regional Ethics Committee, Karolinska Institutet (Diarienumber 2016/1613-31/2).

### Statistical analysis

The computations of descriptive statistics as well as the statistical analysis were performed using the SPSS software (v 25.0). All variables were summarized with standard descriptive statistics such as frequency, mean, and standard deviations. All distributions were checked for severe deviations from a normal distribution. Parametric statistics were preferred for the analysis of approximately normally distributed variables. Thus, comparisons between laxity preoperatively, at 6-month follow-up and laxity reduction from preoperatively to postoperatively for primary and revision ACLR were analyzed with an analysis of variance (ANOVA) for repeated measurements. Comparisons between KOOS scales preoperatively, at 1-year follow-up and changes from preoperatively to postoperatively for primary and revision ACLR were also analyzed with an ANOVA for repeated measurements. The relationship between distributions for categorical variables such as stratified postoperative side-to-side laxity difference and frequency of “surgical failures” at primary and revision ACLR were analyzed with Pearson’s *χ*^2^-test. The significance level in all analysis was 5% (two-tailed).

A total of 118 patients were included in the laxity analysis. With an expected correlation of 0.40 between primary and revision ACLR laxities, an effect size (ES) of 0.39—less than medium sized according to Cohen—can be detected with a power of 0.85. An ES of 0.39 corresponds to a change in laxity of 1.1 mm. The number of patients included in the KOOS analysis was 73. The correlations between the KOOS scales before surgery at primary and revision ACLR varied between 0.17 (Symptoms) and 0.40 (Sport). With a 5 percent significance level and 85% power, a medium effect size, i.e. 0.50, could be detected. This corresponds to a difference of 10 and 14 points for the symptoms and sport subscales, respectively.

## Results

**Fig. 1:**
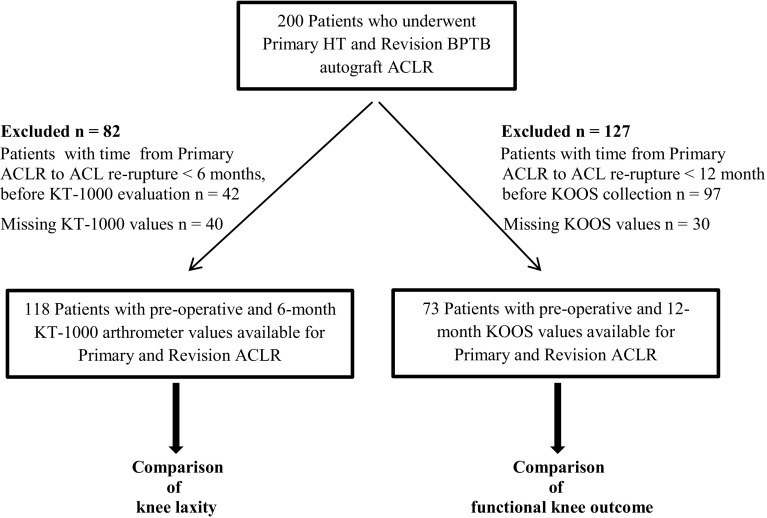
Patient flowchart. The exclusion criteria that led to the final analysis cohort groups are showed. *HT* hamstring tendons, *BPTB* bone–patellar tendon–bone, *ACLR* anterior cruciate ligament reconstruction, *KOOS* Knee Injury and Osteoarthritis Outcome Score

A cohort of 118 patients had preoperative and 6-month postoperative arthrometric values available for both surgeries for the comparison of anterior knee laxity between primary and revision ACLR. A second cohort of 73 patients had preoperative and 1-year postoperative KOOS values available for both surgeries for the comparison of functional knee outcome between primary and revision ACLR (Fig. 1). The demographic data for each cohort are presented in detail in Tables 1 and 2. The total amount of chondral injuries and associated meniscal procedures performed during primary and revision ACLR was higher compared with primary ACLR (Tables 1, 2).

**Table 1:** Patient Demographics (Knee laxity cohort)

Variable		Primary ACLR	Revision ACLR	Total*
Sex, male/female, *n* (%)	60/58 (51.0/49.0)			
Injured side, right/left	64/54			
Age at surgery, y ± SD		21.7 ± 7.1	24.3 ± 7.5	
Cause				
Soccer		57 (48.3)	46 (39.0)	
Alpine skiing		19 (16.1)	17 (14.4)	
Floorball		13 (11.0)	10 (8.5)	
Handball		8 (6.8)	6 (5.1)	
Other sports		13 (11.0)	26 (22.0)	
Other		8 (6.8)	13 (11.0)	
Associated procedures, *n* (%)				
MM resection		19 (16.0)	7 (5.9)	26 (21.9)
LM resection		14 (11.7)	11 (9.2)	25 (20.9)
MM repair		3 (2.5)	8 (6.7)	11 (9.2)
LM repair		6 (5.0)	4 (3.3)	10 (8.3)
MM repair + LM resection		3 (2.5)	1 (0.8)	4 (3.3)
MM resection + LM resection		1 (0.8)	1 (0.8)	2 (1.6)
MM repair + LM repair		1 (0.8)	1 (0.8)	
Chondral lesions, *n* (%)		16 (13.6)	22 (18.6)	38 (32.2)
Fixation methods, *n* (%)				
Femur				
Endobutton		118 (100)	67 (56.8)	
Interference screw		/	51 (43.2)	
Tibia				
AO screw with washer		110 (93.2)	/	
Interference screw		8 (6.8)	118 (100)	
Mean time intervals for primary and revision ACLR, months (range)				
From injury to primary ACLR	7.2 (0.5–74.8)			
From primary ACLR to ACL graft rupture	22.7 (6.5–82.0)			
From ACL graft rupture to revision ACLR	9.0 (1.0–80.2)			

*ACLR* anterior cruciate ligament reconstruction, *ACL* anterior cruciate ligament, *SD* standard deviation, *MM* medial meniscus, *LM* lateral meniscus

*Total amount of meniscal procedures performed and chondral lesions found at primary and revision ACLR

**Table 2:** Patient Demographics (Functional knee outcome cohort)

Variable		Primary ACLR	Revision ACLR	Total*
Sex, male/female, *n* (%)	40/33 (54.7/44.3)			
Injured side, right/left	42/31			
Age at surgery, y ± SD		21.6 ± 7.0	24.7 ± 7.3	
Cause				
Soccer		33 (45.2)	23 (31.5)	
Alpine skiing		12 (16.4)	11 (15.0)	
Floorball		10 (13.7)	9 (12.3)	
Handball		4 (5.5)	3 (4.2)	
Other sport		10 (13.7)	18 (24.7)	
Other		4 (5.5)	9 (12.3)	
Associated procedures, *n* (%)				
MM resection		10 (13.5)	5 (6.7)	15 (20.2)
LM resection		8 (10.8)	5 (6.7)	13 (17.5)
MM repair		3 (4.0)	3 (4.0)	6 (8.0)
LM repair		2 (2.7)	3 (4.0)	5 (6.7)
MM repair + LM resection		3 (4.0)	1 (1.3)	4 (5.3)
MM resection + LM resection		1 (1.3)	1 (1.3)	2 (2.6)
Chondral lesions, *n* (%)		10 (13.5)	14 (18.9)	24 (32.4)
Fixation methods, *n* (%)				
Femur				
Endobutton		73 (100)	40 (54.8)	
Interference screw		/	33 (45.2)	
Tibia				
AO screw with washer		67 (91.8)	/	
Interference screw		6 (8.2)	73 (100)	
Mean time intervals for primary and revision ACLR, months (range)				
From injury to primary ACLR	5.9 (0.5–35.2)			
From primary ACLR to ACL graft rupture	29.9 (13.0–82.6)			
From ACL graft rupture to revision ACLR	8.2 (0.5–48.0)			

*ACLR* anterior cruciate ligament reconstruction, *ACL* anterior cruciate ligament, *SD* standard deviation, *MM* medial meniscus, *LM* lateral meniscus

*Total amount of meniscal procedures performed and chondral lesions found at primary and revision ACLR.

### Knee laxity

**Fig. 2:**
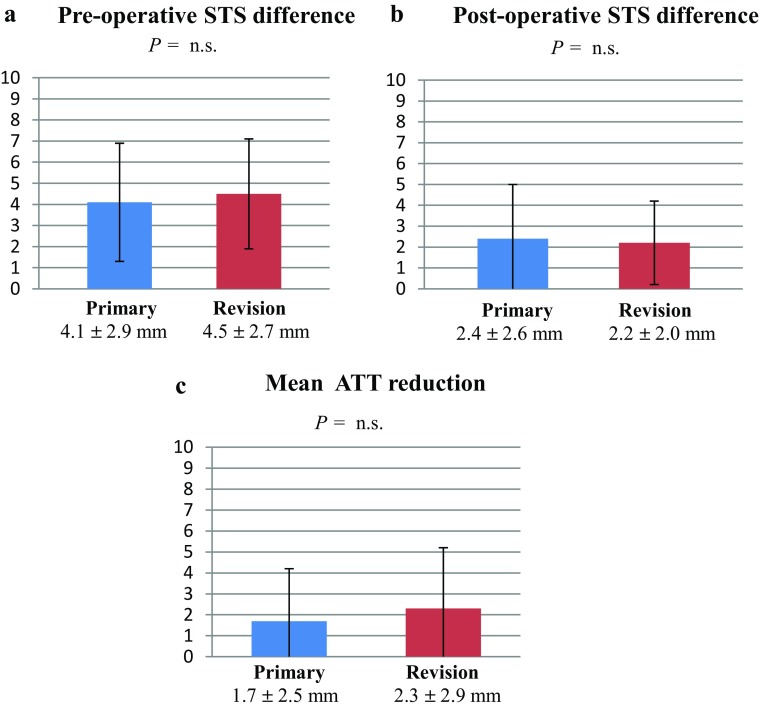
**a–c** Mean anterior STS difference and anterior tibial translation reduction measurements. *STS* side-to-side, *ATT* anterior tibial translation

The mean preoperative and postoperative anterior STS difference as well as the mean anterior tibial translation (ATT) reduction from preoperative to postoperative for the ACL-reconstructed knee were not significantly different between primary and revision ACLR (Fig. 2a–c).

However, primary ACLR showed a significantly higher frequency of postoperative anterior STS difference greater than 5 mm (surgical failure) compared with revision ACLR (Table 3).

**Table 3:** Stratified KT-1000 arthrometer side-to-side difference values

	Number of patients (percentage)		
	≤ 2 mm	3–5 mm	> 5 mm (surgical failures)
Primary ACLR	55 (46.7%)	53 (44.9%)	10 (8.4%)*
Revision ACLR	68 (57.7%)	44 (37.3%)	6 (5.0%)

*A significant higher frequency of “Surgical failures” was found for primary ACLR (*P* = 0.02) compared with revision ACLR

*ACLR* anterior cruciate ligament reconstruction

### Functional knee outcome

#### Preoperative comparison

**Fig. 3:**
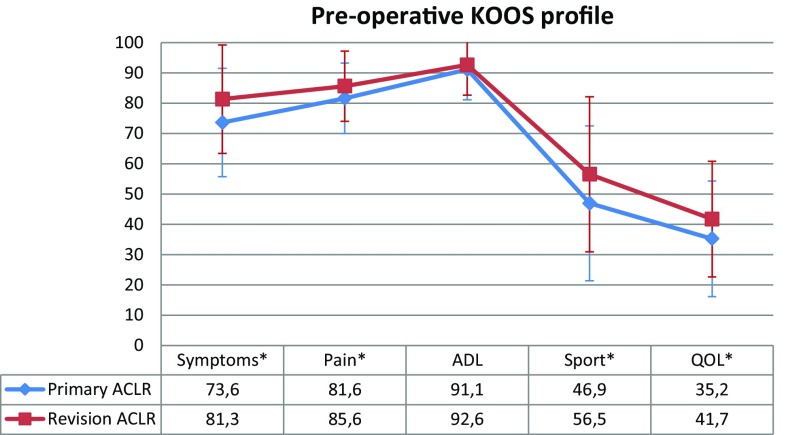
Mean preoperative scores and standard deviation per KOOS subscales for primary and revision ACLR. *KOOS* Knee injury and osteoarthritis outcome score, *ACLR* anterior cruciate ligament reconstruction, *ADL* activity of daily living, *QOL* quality of life. *Statistically significant

The preoperative KOOS values showed significant differences in favour of revision ACLR compared with primary ACLR for the Symptoms (*P* = 0.003), Pain (*P* = 0.01), Sport (*P* = 0.006) and QOL (*P* = 0.04) subscales. No significant difference was found for the ADL subscale (Fig. 3).

#### Preoperative to postoperative comparison

**Fig. 4:**
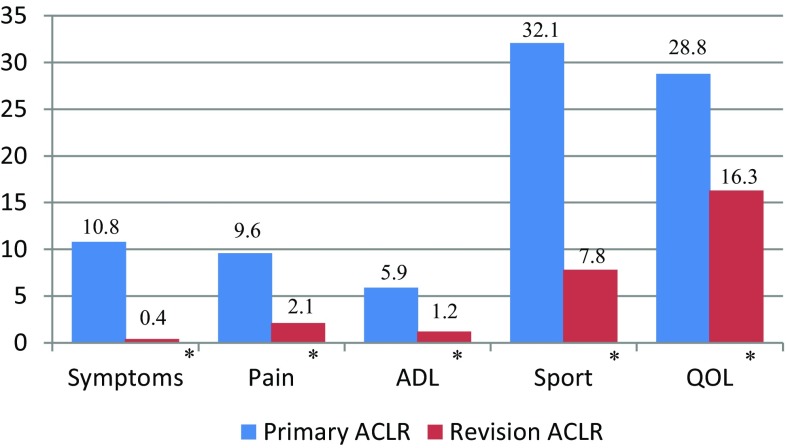
Mean improvement from preoperative to 1-year follow-up per KOOS subscales for primary and revision ACLR. *KOOS* Knee injury and osteoarthritis outcome score, *ACLR* anterior cruciate ligament reconstruction, *ADL* activity of daily living, *QOL* quality of life. *Statistically significant

The mean improvement from preoperative to 1-year follow-up was significantly greater in all KOOS subscales for primary ACLR compared with revision ACLR: Symptoms (*P* = 0.001); Pain (*P* < 0.001); ADL (*P* = 0.002); Sport (*P* < 0.001); QOL (*P* = 0.006) (Fig. 4).

#### Postoperative comparison

**Fig. 5:**
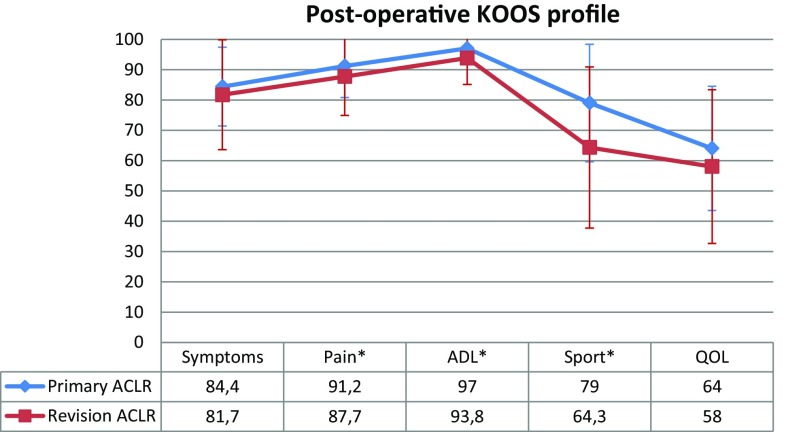
Mean postoperative scores and standard deviation per KOOS subscales for primary and revision ACLR. *KOOS* Knee Injury and Osteoarthritis Outcome Score, *ACLR* anterior cruciate ligament reconstruction, *ADL* activity of daily living, *QOL* quality of life. *Statistically significant

The postoperative KOOS showed significantly better results in Pain (*P* = 0.04), ADL (*P* = 0.003) and Sport (*P* < 0.001) subscales for primary ACLR compared with revision ACLR. No significant differences were found for the Symptoms and QOL subscales (Fig. 5).

## Discussion

The most important finding of this study was that revision BPTB autograft ACLR restores anterior knee laxity but results in inferior functional knee outcome compared with primary HT autograft ACLR in the same cohort of patients.

Our results are in line with previous studies that reported no difference in mean postoperative anterior knee laxity between primary and revision ACLR [1, 6, 16, 20, 36, 37]. However, after primary ACLR we found a higher rate of “surgical failures”, defined as a postoperative anterior STS laxity of more than 5 mm [3, 12]. A recent meta-analysis by Grassi et al. [10] found no differences in the proportion of patients with > 5 mm STS laxity between primary and revision ACLR. The reason for the contrasting results might be that all the revision ACLRs were performed using the BPTB autograft in the present study. This graft could be superior to the HT autograft in restoring anterior knee laxity in the ACL deficient knee [7]. The total amount of chondral lesions and meniscal resections was higher after revision ACLR compared with primary ACLR. This is consistent with previous literature [25, 27]. It has been shown that the meniscus is an important secondary knee stabilizer [2]. Thus, there was a potentially higher risk for greater knee laxity after revision ACLR, due to the loss of secondary restrainers. However, the BPTB graft offers rigid fixation and rapid osteo-integration with its bone plugs. Conversely, the tendon-to-bone healing and slower ligamentization process are characteristics of the HT graft [8, 24]. The higher rate of postoperative anterior STS laxity > 5 mm that we found in primary ACLR, performed with HT autograft, could be a clinical expression of the different biomechanical and biological properties of the grafts used. However, more “surgical failures” (STS laxity > 5 mm) after primary ACLR might also be a reason for a higher risk of graft rupture and need for revision ACLR. Perhaps, these patients were at higher risk for developing a graft rupture and they could not be a representative sample of the entire primary HT autograft ACLR cohort in our database.

The KOOS has been validated to determine the functional outcome in patients with knee injuries and osteoarthritis [32, 33]; however, few studies have used the KOOS to compare the results between primary and revision ACLR [6, 16, 20, 38]. In addition, these studies are based on a matched group analysis or have included different graft types. The Pain, Sport and Recreation and Quality of Life subscales have been indicated to be the most sensitive for changes in the condition of the knee [33]. In the present study, a significantly larger improvement from preoperative to postoperative in all KOOS subscales was found for primary ACLR compared with revision ACLR. In addition, higher scores for Pain, Activity of Daily Living and Sport subscales were found after primary ACLR at 1-year follow-up. However, only the Sport subscale, with a mean difference of 14.7 points between primary and revision ACLR, might represent a clinically significant difference at follow-up. The minimal important difference in KOOS is often considered to be 8–10 points for all subscales [31, 34]. All other subscales, at follow-up, had differences less than 8 points and even if statistically significant the clinical relevance is doubtful. It can be concluded that a satisfactory postoperative outcome can be achieved after both primary and revision ACLR.

Interestingly, preoperative KOOS scores were significantly higher for revision ACLR than for primary ACLR, except for the Activity of Daily Living subscale. Similarly, Weiler et al. [37] found a significantly better preoperative overall Lysholm score for revision ACLR than for primary ACLR. The ACL re-injury probably has less impact on the perceived life situation than the first ACL injury. The higher preoperative KOOS values for revision ACLR could also partly explain the inferior improvement registered in all subscales for this surgery than for primary ACLR. Patients undergoing revision ACLR could have less room for improvement compared with primary ACLR.

Even if, to our knowledge, this is the first study comparing the results between primary and revision ACLR in the same cohort of patients, our findings are in line with previous studies showing that revision ACLR is associated with an inferior functional knee outcome compared with primary ACLR [1, 6, 10, 16, 20, 26, 37, 38].

There are several potential reasons for the worse postoperative functional outcome after revision ACLR in comparison with primary ACLR, such as more chondral and meniscal lesions, increased pain due to the repeated surgical trauma, the patients have had two serious knee injuries (ACL tear and ACL graft rupture) as well as multiple graft harvesting with impairment of both flexor and extensor knee mechanism [10, 37]. Another explanation for the inferior functional results after revision ACLR in the present study could be the potential increased “donor morbidity site” associated with BPTB autograft compared with HT autograft [5, 21, 39]. Tomihara et al. [36], in a recent matched group analysis, showed that revision BPTB autograft ACLR provides compatible postoperative clinical outcomes and knee stability compared with primary BPTB autograft ACLR. In the present study, we compared instead primary HT autograft ACLR with revision BPTB autograft ACLR. Thus, there could be also a significant effect of graft choice for the differences in the functional knee outcome that we found.

This study shows that revision ACLR using BPTB autograft after failure of primary HT autograft ACLR restores joint laxity, and consequently may protect the knee against further meniscal and cartilage injuries as suggested for primary ACLR [15, 17]. Salmon et al. [35] found that articular surface damage is associated with the chronicity of a failed primary ACLR, suggesting to perform revision ACLR in the sub-acute phase before that more episodes of giving-way occur. However, patients must be aware of the fact that having revision ACLR their knee function will not improve as much as with primary ACLR and the final postoperative functional outcome is inferior. It has been shown that patients usually have very high demands and perhaps unrealistic expectations regarding ACLR, and this can negatively influence patient-reported outcomes causing patient dissatisfaction [4].

The main strength of this study was that the same cohort of patients who underwent primary and revision ACLR was directly compared. All patients received surgery, rehabilitation, preoperative and postoperative assessment for both surgeries at the same institution. Moreover, only one type of graft has been used for all primary (HT autograft) and revision (BPTB autograft) ACLR.

The main limitation is the short-term follow-up. However, since most primary ACL graft failures occurred within two postoperative years, a study based on the same cohort of patients with longer follow-up would be difficult to perform. Another limitation is that we had no information regarding the severity of the chondral lesions and the extent and location of meniscal resection or repair. No data to compare the return to sport rate between primary and revision ACLR were available. Finally, another limitation is the lack of information regarding rotational laxity, which has been associated with the subjective outcome [18].

## Conclusions

The findings of this study showed that anterior knee laxity is restored with revision BPTB autograft ACLR after failed primary HT autograft ACLR, in the same cohort of patients. However, revision ACLR showed significantly inferior functional knee outcome compared with primary ACLR. It is important for clinicians to inform and set realistic expectations for patients undergoing revision ACLR. Patients must be aware of the fact that having revision ACLR their knee function will not improve as much as with primary ACLR and the final postoperative functional outcome is inferior.
